# Role of CTRP3, CTRP9 and MCP-1 for the evaluation of T2DM associated coronary artery disease in Egyptian postmenopausal females

**DOI:** 10.1371/journal.pone.0208038

**Published:** 2018-12-17

**Authors:** Sara F. Ahmed, Marwa I. Shabayek, Mostafa E. Abdel Ghany, Mohamed H. El-Hefnawy, Hala O. El-Mesallamy

**Affiliations:** 1 Biochemistry Department, Faculty of Pharmaceutical Sciences and Pharmaceutical Industries, Future University in Egypt, 5th settlement, Cairo, Egypt; 2 Cardiology Department, Faculty of Medicine, Al-Azhar University, Nasr city, Cairo, Egypt; 3 National Institute of Diabetes and Endocrinology (NIDE), Kasr El Ainy, Cairo, Egypt; 4 Biochemistry Department, Faculty of Pharmacy, Ain Shams University, African Union Authority St. Abassia, Cairo, Egypt; East Tennessee State University, UNITED STATES

## Abstract

C1q complement/tumor necrosis factor (TNF)—related protein (CTRP) family comprises of 15 proteins that posses important implications in energy homeostasis, infection and inflammation. However, their roles in diabetes mellitus (DM) and its vascular complications have not been completely assessed. This works aims to study the association of two CTRPs; 3 and 9, with pro-inflammatory cytokine monocyte chemoattractant protein-1 (MCP-1), and biochemical parameters of type 2 diabetes (T2D), dyslipidemia and coronary artery disease (CAD).**Methods**: Biochemical markers and serum levels of CTRPs and MCP-1 were measured in 86 postmenopausal females. Subjects were divided over four groups; 13 apparent healthy subjects as control (group I), 29 patients with CAD (group II), 29 patients with T2D ≥5 years (group III) and 15 patients with CAD secondary to T2D (group IV). Serum CTRP3, CTRP9, MCP-1 and insulin were measured by ELISA.**Results**: Serum CTRP3 levels were found to be significantly higher in group III and IV, whereas, it was significantly lower in group II on comparing to group I. While, CTRP9 levels were significantly decreased in group II, III and IV on comparing to group I. MCP-1 levels were found to be significantly increased in groups II, III and IV on comparison with group I. Both CTRPs were significantly negatively correlated with each other. While MCP-1 was significantly correlated negatively to CTRP9.**Conclusion**: This study associates the possible role of CTRP3, CTRP9 and MCP-1/CCL2 in the diagnosis/prognosis of CAD complication in T2D postmenopausal females.

## Introduction

Diabetes mellitus (DM) is a chronic metabolic disease, its development involves several pathogenic processes [1]. Collectively all of these processes results in loss of β-cell mass and/or function that are clinically manifested as hyperglycemia [2]. The number of diabetic patients in 2015 were estimated to be around 415 million people and expected to reach 642 million by the year 2040, which represents an exponential increase mainly in developing countries[[3].

Among the several types of DM, type 2 diabetes (T2D) is a progressive pandemic that accounts for more than 90% of all cases, representing an indisputable threat to the public health[[4]. T2D is a complex polygenic disorder which is a result of the combination of hereditary components and secondary contributing factors which include; obesity, lifestyle, birth weight and stress [5].

Obesity represents one of the prominent risk factors for T2D and its complications, it shows an equivalent increase in its global prevalence [6]. Visceral obesity and adipose tissue causes, metabolic disturbance, leading to various obesity associated co-morbidities including; metabolic syndrome, T2D, hyperlipidemia and heart diseases [7]. In addition, obesity is correlated with T2D through proinflammatory cytokines (adipokines), insulin sensitivity, abnormal fatty acid metabolism, and intracellular disturbances; mitochondrial dysfunction and endoplasmic reticulum stress [8,9].

In 2004, the CTRP super-family emerged as novel anti-inflammatory adipokines with important metabolic roles [10]. The CTRP family is composed of 16 members including adiponectin (APN), like APN, the CTRP family members were believed to play a vital role in energy homeostasis, through altering insulin sensitivity specifically in the muscles and liver, all of which regulates whole-body energy metabolism thus, CTRPs could be novel pharmacological targets in T2D, obesity and inflammatory diseases [11].

A member of the CTRP family and one of the closest functional homolog of APN with a potent anti-inflammatory, antidiabetic and cardioprotective adipokine is CTRP3 (also known as cartonectin, cartducin, CORS-26) [9,10]. Another member is CTRP9, which plays a role in lowering blood glucose levels, with a potent vasorelaxation effect as its vasoactive potency is about three folds higher than that of APN [12]. However, CTRP9 has not been fully investigated in human subjects suffering from DM.

The monocyte chemoattractant protein-1 (MCP-1/CCL2) is a member of the C-C chemokine family, a potent chemotactic factor for monocytes with potent proinflammatory action attributing to many inflammation mediated diseases, such as T2D and coronary artery disease (CAD) [13]. Interestingly, the proinflammatory action of MCP-1/CCL2 was found to be blocked by CTRP3 [11]. However the relationship between the anti-flammatory adipokine CTRP9 and proinflammatory chemokine MCP-1/CCL2 has not been studied along with its role in T2D and CAD.

The current study was designed to develop effective diagnostic and prognostic strategies through evaluating the changes in the levels of CTRP3, CTRP9 and MCP-1/CCL2 in females suffering from either T2D only, CAD only, and CAD secondary to T2D. In addition, correlation between CTRP3, CTRP9, MCP-1/CCL2 with each other and with other routine biochemical parameters were assessed to explain how these possible markers correlate with the diagnosis/prognosis of the patients suffering from T2D alone, CAD alone and the development of CAD in T2D patients.

## Material and methods

### Subjects

This study was approved by the Medical ethics committee of Ain shams University, a signed informed consent was obtained from all subjects prior to participation in the study. The study complies with all the regulations and recommendations of the declaration of Helsinki.

About 86 subjects were divided into four groups, as follows: Group I included (13) apparent healthy subjects as control with average body mass index (BMI) (**26.33±0.25)**. Group II included (29) patients with CAD with average BMI (**29.89±0.10)**. Group III included (29) patients with T2D ≥ 5 years with average BMI (**29.50±0.05)**, group IV included (15) patients with CAD secondary to T2D with average BMI (**30.46±0.19)**. The range of BMI between: 18.50–24.99, was classified as normal. While BMI 25–29.99 kg/m^2^ was classified as overweight (non obese) subjects, and BMI ≥ 30 kg/m^2^ was classified as obese subjects[14]. Blood samples were withdrawn from patients in the National Institute of Diabetes and Endocrinology (NIDE), (Cairo, Egypt) and the intensive care unit (ICU) of the cardiology department of Al-Hussein Teaching Hospital (Cairo, Egypt). All the subjects are postmenopausal females. Characteristics of subjects are represented in Table 1.

**Table 1:** Clinical and laboratory characteristics of the studied groups.

Groups/Parameters	Group(I) (n = 13)	Group(II) (n = 29)	Group(III) (n = 29)	Group(IV) (n = 15)
**Age (Years)**	**51.15±1.82**	**56.10±0.80**	**50.48±1.38**^b^	**56.67±1.63**^c^
**BMI (kg/m**^**2)**^	**26.33±0.25**	**29.89±0.10**^a^	**29.50±0.05**^a^	**30.46±0.19**^a^^,^^b^^,^^c^
**FBG (mg/dl)**	**97.46±2.37**	**96.45±1.54**	**197.79±3.22**^a^^,^^b^	**189.33±4.26**^a^^.^^b^
**Hb**_**A1**_**c %**	**5.15±0.08**	**5.176±0.07**	**8.476±0.15**^a^^,^^b^	**8.73±0.21**^a^^**,**^^b^
**Insulin (pmol/l)**	**7.31±0.62**	**9.17±1.83** ^a^	**13.69±1.28** ^a^^,^^b^	**14.80±0.76**_a_
**Duration of T2D (years)**	**————**	**——————**	**7.09±0.29**	**8.40±0.46**^c^
**HOMA-IR**	**1.69±0.13**	**4.52±0.41** ^a^	**6.69±0.62** ^a^^,^^b^	**7.00±0.48** ^a^^,^^b^
**QUICKI**	**0.35±0.01**	**0.31±0.01** ^a^	**0.30±0.004** ^a^	**0.29±0.002** ^a^^,^^b^
**TC (mg/dl)**	**173.38±3.19**	**237.55±4.52**^a^	**257.69±4.76**^a^^,^^b^	**271.67±8.07**^a^^,^^c^
**TG (mg/dl)**	**143.62±1.82**	**179.2±1.66** ^a^	**178.66±1.89** ^a^	**191.60±4.68** ^a^^,^^b^^,^^c^
**LDL-C (mg/dl)**	**99.77±1.48**	**182.83±2.09** ^a^	**181.14±1.99** ^a^	**191.73±2.75** ^a^^,^ ^b^^,^^c^
**HDL-C (mg/dl)**	**52.33±1.78**	**39.31±0.70** ^a^	**38.41±0.77** ^a^	**28.93±1.07** ^a^^,^ ^b^^,^ ^c^
**LDL-C/HDL-C ratio**	**1.92±0.08**	**4.62±0.10** ^a^	**4.76±0.11** ^a^	**6.73±0.33** ^a^^,^ ^b^^,^ ^c^
**TC/HDL-C ratio**	**3.38±0.14**	**6.10±0.15** ^a^	**6.69±0.13** ^a^	**9.53±0.44** _a__,_ _b__,_ _c_
**CTRP3 (ng/ml)**	**130.38±7.12**	**80.31±3.05**^a^	**211.34±5.75**^a^^,^^b^	**186.93±4.02**^a^^,^^b^^,^^c^
**CTRP9 (ng/ml)**	**304.46±9.60**	**194.90±3.34**^a^	**126.76±2.95**^a^^,^^b^	**101.40±5.70** ^a^^,^^b^^,^^c^
**MCP-1(pg/ml)**	**190.54±6.12**	**469.45±6.12**^a^	**331.86±11.41**^a^^,^^b^	**676.27±28.37** ^a^^,^^b^^,^^c^

Values are expressed in terms of (Mean ± SEM).

Group I: Apparent healthy control; Group II: patients with CAD; Group III: Patients with type 2 diabetes; Group IV: patients with CAD secondary to type 2 diabetes.

BMI: body mass index; FBG: fasting blood glucose; HbA1c: glycated hemoglobin; TG: triglycerides; TC: total cholesterol; LDL-c: low density lipoprotein cholesterol; HDL-c: high density lipoprotein cholesterol; HOMA-IR: homeostasis model assessment—Insulin resistance; QUICKI—quantitative insulin sensitivity check index

^a^: Significantly different from control group I at p<0.05

^b^: Significantly different from group II at p<0.05

^c^: Significantly different from group III at p<0.05

Medical history was collected from all subjects including; duration of DM (at least 5 years), familial history of DM, type of CAD, medication list, history of any disease and surgical procedures. All subjects were physically examined. CAD was previously diagnosed using cardiac angiogram. All diabetic patients were treated using 500mg metformin twice daily.

The following exclusion criteria were used for all study participants: All patients were free of chronic liver diseases, type 1 DM (T1D), acute or chronic renal disease, hyperthyroidism, disorders in pituitary gland, malignancy, autoimmune disease, and inflammatory diseases. Patients were not taking any anti-inflammatory drugs, as well as other medications that may affect the heart.

### Methods

#### Blood sampling

Whole blood samples were collected after an overnight fast from all 86 subjects. Blood collected was split into three portions; the first portion was used to measure fasting plasma glucose (FPG) and collected in Na fluoride containing vacutainer tubes. The second portion was collected on EDTA containing vacutainer tubes in order to measure glycated hemoglobin (HbA_1_c %). The third portion of blood was centrifuged to separate serum for the measurement of: insulin, lipids [triglycerides (TG), total cholesterol (TC), low density lipoprotein cholesterol (LDL-C), high density lipoprotein cholesterol (HDL-C)] and the serum levels of CTRP3, CTRP9 and MCP-1. All routine work analyses were measured in the same day of the blood collection, the remaining samples were stored at -80°C till time of assay for insulin, CTRP3, CTRP9 and MCP-1.

#### Laboratory assessment

Patient’s demographics and medical history were obtained from the patients file or during the patients visit. BMI was calculated using the standard formula (weight (kg)/height (m^2^)). Insulin level was measure using BioTina GmbH, Germany ELISA kit (Cat No: E 2035). Levels of FBG (Cat. No: GL 13 20), Hb_A1_c% (Cat. No: k031296) and levels of TG (Cat. No: TR 20 30), TC (Cat. No: CH 12 20), HDL-C (Cat. No: CH 12 30),and LDL-C (Cat. No: CH 12 31) were measured using Biodiagnostic Co., Egypt kits. The homeostasis model assessment of insulin resistance (HOMA-IR) was calculated using the following equation: [fasting blood glucose (mg/dL)] × [fasting blood insulin (μU/mL)/405]. For calculating Quantitative Insulin Sensitivity Check Index we used the following equation (QUICKI: 1/(ln (insulin)+ln (glucose)). The serum levels of CTRP3, CTRP9 and MCP-1 were measured using Aviscera biosciences, USA ELISA kits (Cat. No: SK00082-07 for CTRP3, SK00081-02 for CTRP9 and SK00220-01 for MCp-1).

### Statistical analysis

All results were expressed as mean ± standard error of mean (M ± S.E.M). Analysis of variance (ANOVA) was used to compare different groups and to compare individual groups post Hoc LSD was applied. Kolmogorov smirnov test was conducted to ensure the normal distribution of the data and our data met the null hypothesis of the normal distribution. The correlation between the parameters was done using Pearson correlation test which is an extended parametric analysis Simple and multiple linear stepwise regression analyses were done to study the association between each of CTRP3, CTRP9 and MCP-1 with other biochemical parameters. In the multiple linear stepwise regression analysis, the independent variables included demographic factors and other biochemical variables (BMI, age, duration of diabetes, FBG, Hb_A1_c %, insulin, TC, TG, HDL-C, LDL-C, TC/ HDL-C risk ratio, HDL-C /LDL-C risk ratio, HOMA-IR and QUICKI), all of which were associated with the examined dependent variable (CTRP3, CTRP9 and MCP-1/CCL2) in univariate analysis. Windows based SPSS statistical package (SPSS version 20.0, SPSS Inc, Chicago, IL) was used to perform all statistical analyses. *P*-values < 0.05 were considered significant.

## Results

The power analysis done to power our study, used CTRP3 level as the primary outcome. The effect size d = (2.96975) was calculated based upon the results of Choi et al (2012) for the difference between normal and T2D subjects[15]. The effect size (d) was converted to effect size (f = 1.4849) using effect size converter designed by Jamie DeCoster (2012) to allow for calculating sample size for comparison between the four groups because there was no relevant studies to the present study. Using alpha (α) level of (5%) and Beta (β) level of (20%) i.e. power = 80%; the minimum estimated sample size was 12 subjects per group. Sample size calculation was performed using G*Power Version 3.1.9.2.

The clinical characteristics and serum levels of CTRP3, 9 and MCP-1of the studied groups are shown in Table 1. BMI, FBG, Hb_A1_c %, insulin, HOMA-IR, QUICKI and all lipids profile parameters showed higher levels in groups II, III and IV as compared to control group except HDL-C and QUICKI.

**Fig 1:**
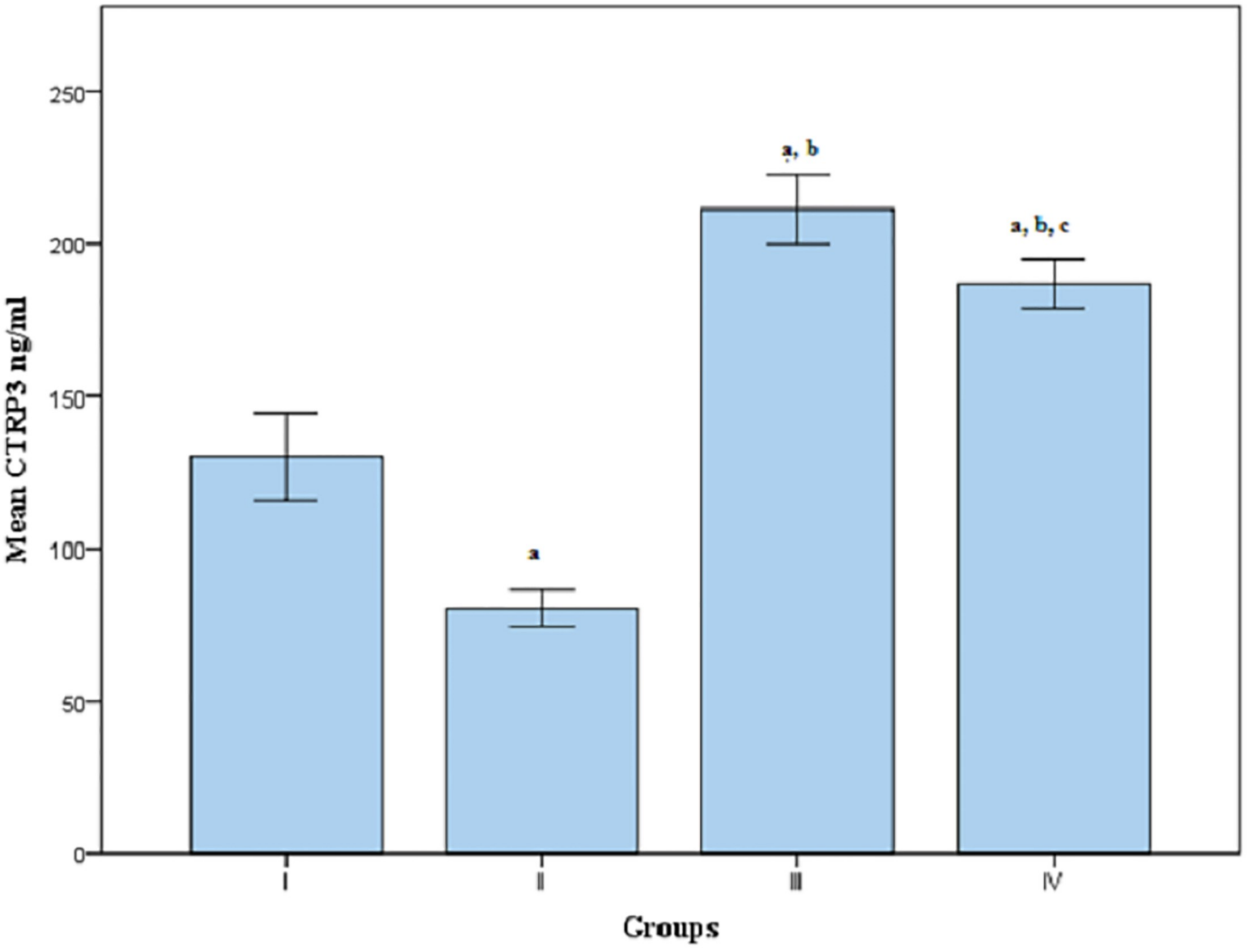
Mean serum CTRP3 levels in groups I, II, III and IV. Group(I):Apparent healthy control, Group(II):Patients with CAD, Group(III):Patients with T2DM, Group(IV):Patients with CAD secondary to T2DM, a: Significantly different from group I at p<0.05, b: Significantly different from group II at p<0.05, c: Significantly different from group III at p<0.05, CTRP3: C1q complement/tumor necrosis factor (TNF)—related protein 3.

Serum CTRP3 level was found to be significantly higher in group III and group IV whereas, it was significantly lower in group II when compared to group I. In addition, its levels were significantly decreased in group IV when compared to group III. Moreover, CTRP3 level was further elevated significantly in group III when compared to group II at *p*<0.05[Table 1, Fig 1].

**Fig 2:**
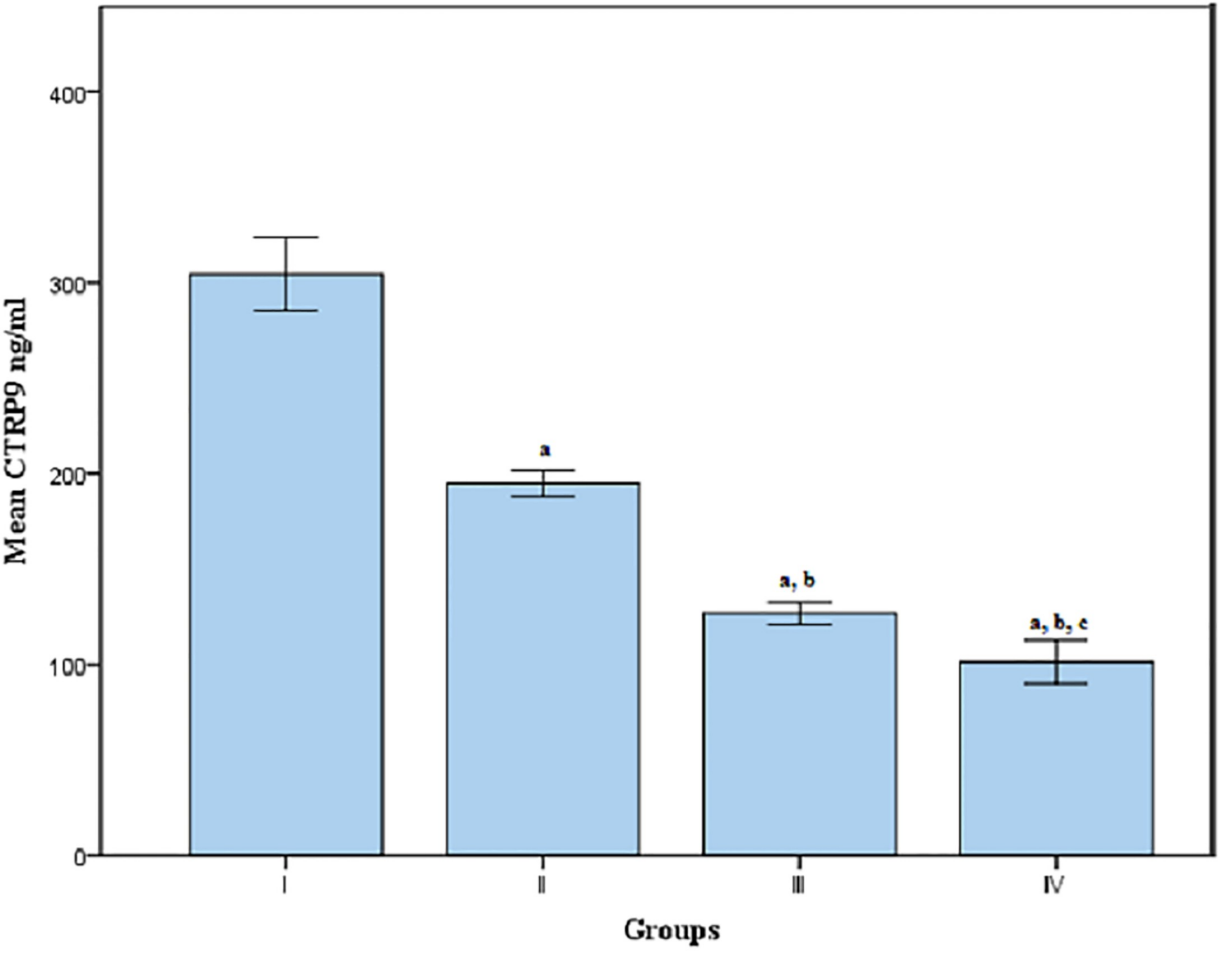
Mean serum CTRP9 levels in groups I, II, III and IV. Group(I):Apparent healthy control, Group(II):Patients with CAD, Group(III):Patients with T2DM, Group(IV):Patients with CAD secondary to T2DM, a: Significantly different from group I at p<0.05, b: Significantly different from group II at p<0.05, c: Significantly different from group III at p<0.05, CTRP9: C1q complement/tumor necrosis factor (TNF)—related protein 9.

Regarding serum CTRP9 level, it was found to be significantly decreased in group II, III and IV when compared to group I. Meanwhile, its level was significantly decreased in group IV, when compared to group II and III. Moreover, CTRP9 level was significantly decreased in group III when compared to group II at p<0.05[Table 1, Fig 2].

**Fig 3:**
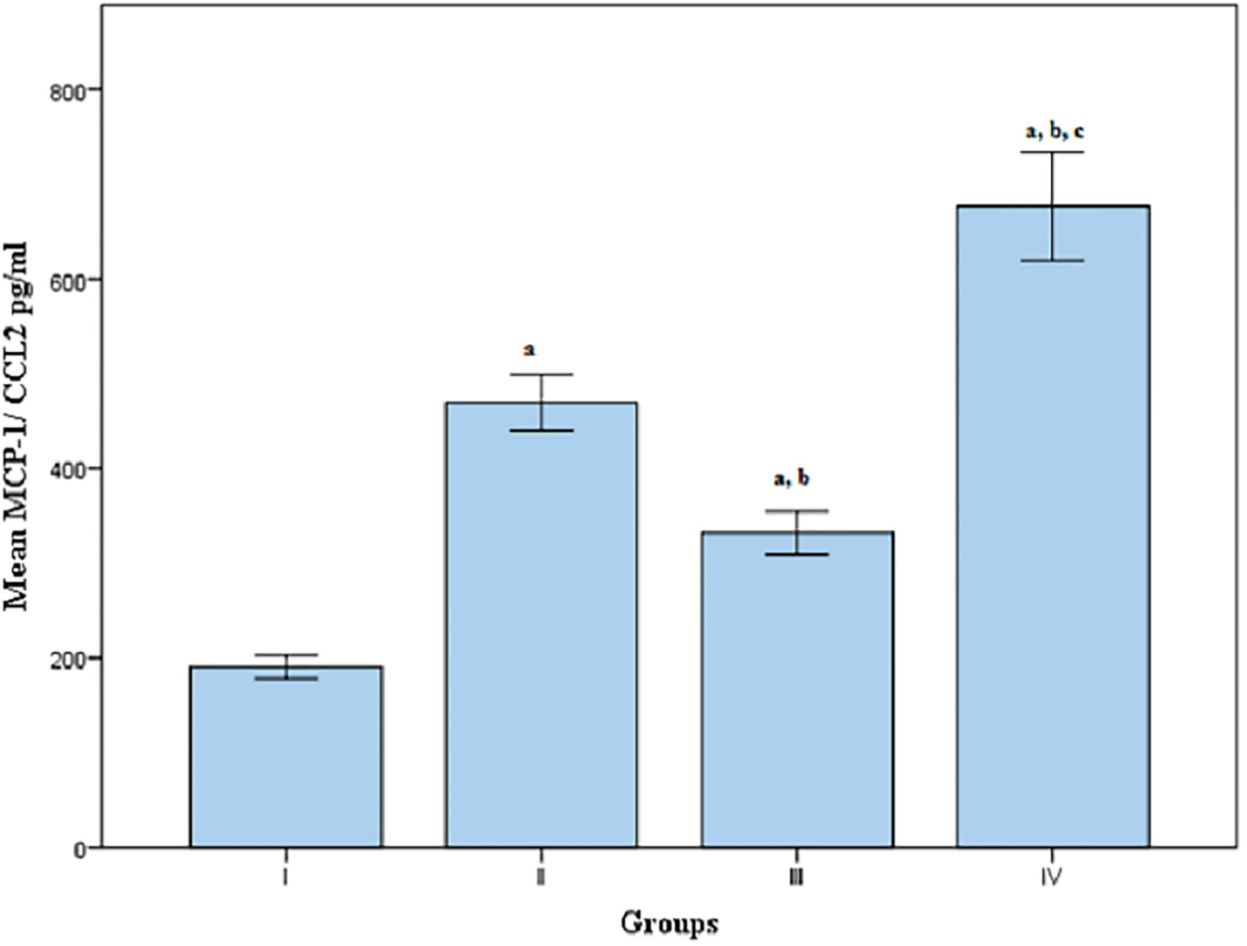
Mean serum MCP-1/ CCL2 3 levels in groups I, II, III and IV. Group(I):Apparent healthy control, Group(II):Patients with CAD, Group(III):Patients with T2DM, Group(IV):Patients with CAD secondary to T2DM, a: Significantly different from group I at p<0.05, b: Significantly different from group II at p<0.05, c: Significantly different from group III at p<0.05, MCP-1: Monocyte chemoattractant protein-1.

Regarding serum MCP-1 level, it was found to be significantly increased in group II, III and IV when compared to group I. Meanwhile, its level was significantly increased in group IV, when compared to group II and III. Moreover, MCP-1 level was significantly decreased in group III when compared to group II and IV at p<0.05[Table 1, Fig 3].

**Fig 4:**
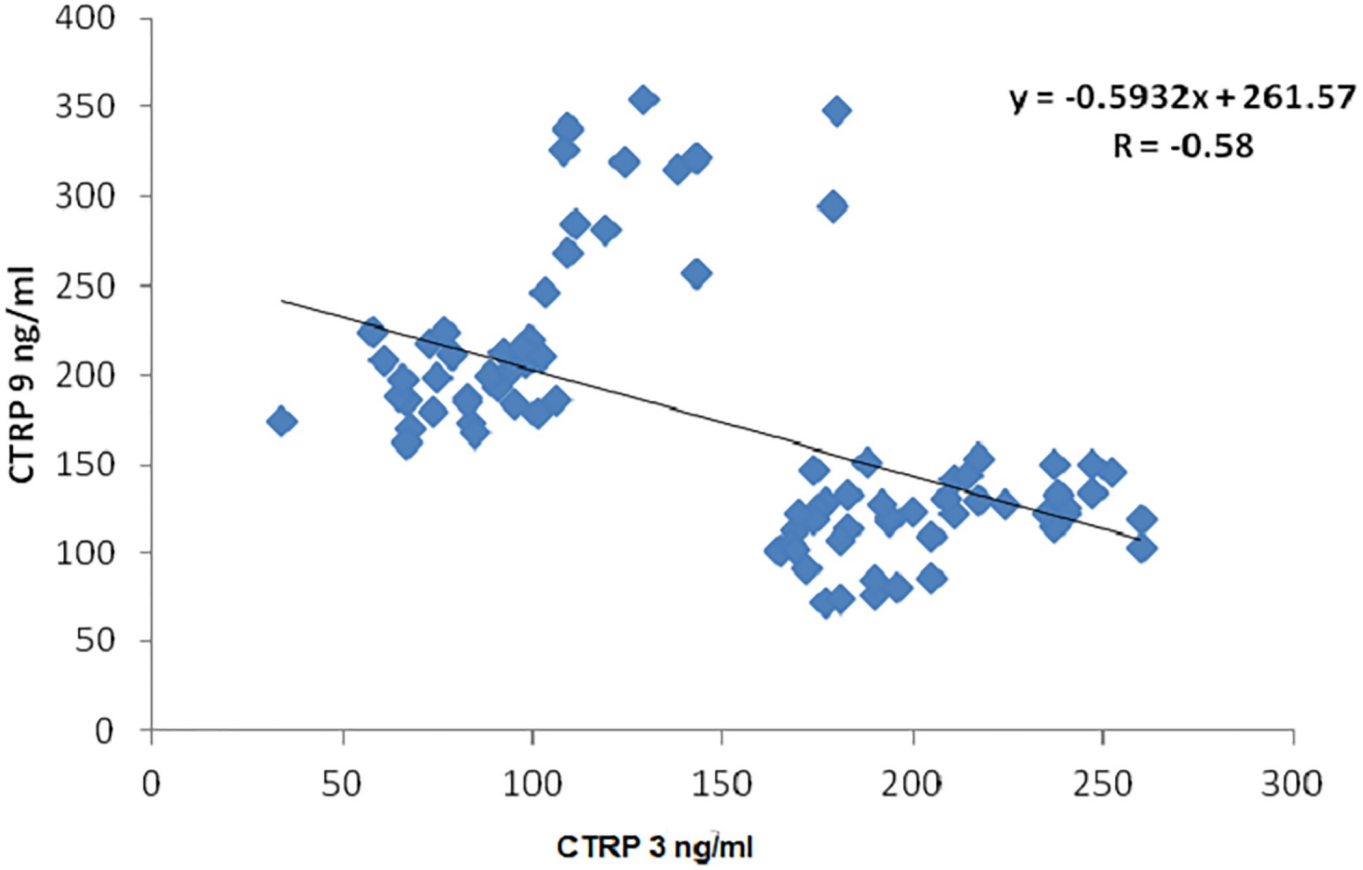
Correlation between CTRP9 and CTRP3. CTRP3: C1q complement/tumor necrosis factor (TNF)—related protein 3, CTRP9: C1q complement/tumor necrosis factor (TNF)—related protein 9.

On performing simple linear regression using CTRP3 as the dependent variable and other biochemical parameters as the independent variables, CTRP3 was found to be significantly positively correlated with other independent variables including; FBG, Hb_A1_c %, T2D duration, TC, TC/HDL-C, and HOMA-IR while, significantly negatively correlated with age, HDL-C, QUICKI and CTRP9 at p<0.05 [Table 2, Fig 4]. While, on performing multiple linear stepwise regression analysis using CTRP3 as dependent variable with other independent variables only FBG (β = 0.276, P = 0.007), Hb_A1_c % (β = 0.413, P = 0.002) and T2D duration (β = 0.384, P = 0.003), remained significantly associated with CTRP3 [Table 3].

**Table 2:** Simple linear regression of CTRP3, CTRP9 and MCP-1 as dependant variables.

Variable	CTRP3		CTRP9		MCP-1	
	β (r)	P	β (r)	P	β (r)	P
**Age (Years)**	-0.24*	**0.02**	**-0.01**	**0.95**	0.32**	**0.01**
**BMI (kg/m**^**2)**^	**0.02**	**0.87**	-0.43**	**0.01**	0.63**	**0.01**
**FBG (mg/dl)**	0.70**	**0.01**	-0.71**	**0.01**	**0.06**	**0.57**
**Hb**_**A1**_**c %**	0.72**	**0.01**	-0.74**	**0.01**	**0.14**	**0.21**
**Insulin (pmol/l)**	**-0.17**	**0.12**	**-0.10**	**0.34**	0.47**	**0.01**
**Duration of T2D (years)**	0.76**	**0.01**	-0.82**	**0.01**	**0.18**	**0.09**
**HOMA-IR**	0.39**	**0.01**	-0.60**	**0.01**	0.41**	**0.01**
**QUICKI**	-0.39**	**0.01**	0.59**	**0.01**	-0.45**	**0.01**
**TC (mg/dl)**	0.37**	**0.01**	-0.62**	**0.01**	0.45**	**0.01**
**LDL-C (mg/dl)**	**0.08**	**0.46**	-0.44**	**0.01**	0.57**	**0.01**
**TG (mg/dl)**	**0.02**	**0.83**	-0.55**	**0.01**	0.50**	**0.01**
**HDL-C (mg/dl)**	-0.23*	**0.04**	0.60**	**0.01**	-0.60**	**0.01**
**LDL-C/HDL-C ratio**	**0.21**	**0.06**	**-0.60**	**0.01**	0.60**	**0.01**
**TC/HDL-C ratio**	0.35**	**0.01**	-0.67**	**0.01**	0.59**	**0.01**
**CTRP3 (ng/ml)**	**——**	**———**	-0.58**	**0.01**	**-0.16**	**0.14**
**CTRP9 (ng/ml)**	-0.58**	**0.01**	**———**	**———**	-0.36**	**0.01**
**MCP-1 (pg/ml)**	**-0.16**	**0.14**	-0.36**	**0.01**	**——**	**——**

**Correlation is significant at the 0.01 level.

*Correlation is significant at the 0.05 level.

BMI: body mass index; FBG: fasting blood glucose; HbA1c: glycated hemoglobin; TG: triglycerides; TC: total cholesterol; LDL-c: low density lipoprotein cholesterol; HDL-c: high density lipoprotein cholesterol; HOMA-IR: homeostasis model assessment -Insulin resistance; QUICKI—quantitative insulin sensitivity check index

**Table 3:** Multiple linear regression CTRP3, CTRP9 and MCP-1 as dependant variables.

Variable	CTRP3		CTRP9		MCP-1	
	β (r)	P	β (r)	P	β (r)	P
**BMI (kg/m**^**2)**^	**———**	**———**	**-0.243**	**0.004**	**0.199**	**0.047**
**FBG (mg/dl)**	**0.276**	**0.007**	**-0.262**	**0.008**	**———**	**———**
**Hb**_**A1**_**c %**	**0.413**	**0.002**	**———**	**———**	**———**	**———**
**Duration of T2D (years)**	**0.384**	**0.003**	**-0.277**	**0.005**	**———**	**———**
**LDL-C (mg/dl)**	**———**	**———**	**-0.215**	**0.012**	**———**	**———**
**TC/HDL-C ratio**	**———**	**———**	**———**	**———**	**0.681**	**0.000**
**CTRP3 (ng/ml)**	**———**	**———**	**———**	**———**	**-0.298**	**0.000**

**Correlation is significant at the 0.01 level.

*Correlation is significant at the 0.05 level.

BMI: body mass index; FBG: fasting blood glucose; HbA1c: glycated hemoglobin; TG: triglycerides; TC: total cholesterol; LDL-c: low density lipoprotein cholesterol; HDL-c: high density lipoprotein cholesterol.

**Fig 5:**
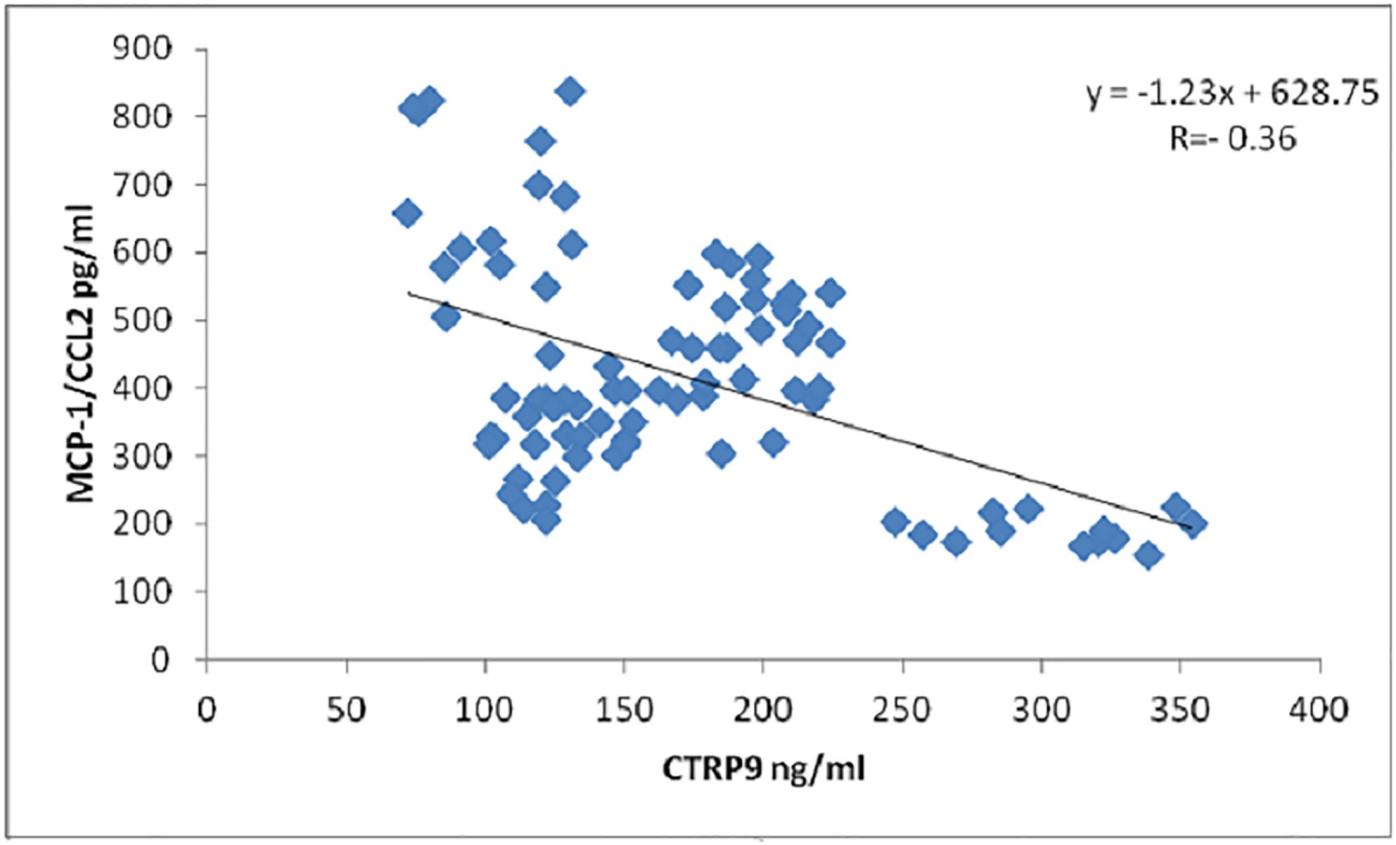
Correlation between MCP-1/ CCL2 and CTRP9. CTRP9: C1q complement/tumor necrosis factor (TNF)—related protein 9, MCP-1/ CCL2: Monocyte chemoattractant protein-1.

On performing simple linear regression using CTRP9 as the dependent variable and other biochemical parameters as the independent variables, CTRP9 was found to be significantly positively correlated with HDL-C and QUICKI while, significantly negatively correlated with other independent variables including; BMI, age, duration of diabetes, FBG, Hb_A1_c %, TC, TG, LDL-C, TC/ HDL-C, HDL-C/LDL-C, HOMA-IR, CTRP3 and MCP-1/CCL2 [Table 2, Figs 4 and 5]. Moreover, on performing multiple linear stepwise regression analysis using CTRP9 as dependent variable with other independent variables only LDL-C (β = -0.215, P = 0.012), FBG (β = -0.262, P = 0.008), BMI (β = -0.243, P = 0.004) and T2D duration (β = -0.277, P = 0.005), remained significantly associated with CTRP9 [Table 3].

On performing simple linear regression using MCP-1 as the dependent variable and other biochemical parameters as the independent variables, MCP-1was found to be significantly positively correlated with other independent variables including; BMI, T2D duration, TC, TG, LDL-C, TC/HDL-C, LDL-C/HDL-C, insulin, and HOMA-IR while, significantly negatively correlated with age, HDL-C, QUICKI and CTRP9 [Table 2, Fig 5]. While, on performing multiple linear stepwise regression analysis using MCP-1/CCL2 as dependent variable with other independent variables only TC/HDL-C (β = 0.681, P = 0.000), CTRP3 (β = -0.298, P = 0.000) and BMI (β = 0.199, P = 0.047) remained significantly associated with MCP-1[Table 3].

## Discussion

C1q complement/TNF-related protein 3 and 9 can be considered critical players in the pathogenesis of T2D, dyslipidemia, and CAD, since their expression is dysregulated in metabolic diseases[16]. Thus, this study was conducted to investigate the correlation between CTRP3, CTRP9 and MCP-1/CCL2 in diabetic postmenopausal females with or without CAD.

In this current study the levels of CTRP3 were found to be positively correlated with hyperglycemia. In addition, its levels were significantly higher in females suffering from T2D only, and those suffering from T2D with CAD. Interestingly, conflicting results were reported from studies investigating the levels of serum CTRP3 in T2D patients and metabolic syndrome. One study showed elevated CTRP3 levels in patients with T2D [17], where as another study showed lower CTRP3 levels in newly diagnosed T2D [18]. In our study CTRP3 proved to be positively associated with DM biomarkers (FBG, HbA1c%, HOMA-IR and T2D duration) and negatively associated with QUICKI and MCP-1/CCL2 indicating its strong clinical association with the development of T2D. To the best of our knowledge this is the first study to investigate the correlation between T2D duration, QUICKI and MCP-1/CCL2 and CTRP3 levels. This correlation proposed that the elevated CTRP3 levels in T2D could be a compensatory mechanism developed in long standing DM to decrease the state of hyperglycemia through altering the increased insulin resistance (IR) and decreased insulin sensitivity (measured by QUICKI) present in these patients. This assumption also concurs with the proposed mechanism by Peterson *et al*. for CTRP3 that in the absence of insulin, CTRP3 suppresses gluconeogenic enzymes expression [glucose-6-phosphatase and phosphoenolpyruvate carboxykinase], resulting in a decreased glucose production in hepatoma cells [19]. The paradoxical increase in CTRP3 levels in T2D females could also be attributed to the effect of metformin, as Tan *et al*. stated that metformin stimulates the production and secretion of CTRP3 from adipose tissue significantly increasing its circulating level [20].

In this study CTRP3 serum levels were decreased in T2D with CAD females in comparison with T2D females and the lowest levels of CTRP3 were observed in female patients with CAD only. Interestingly, in this study CTRP3 exhibited a positive correlation with dyslipidemia biomarkers (TC, TG, LDL-C,TC/ HDL-C risk ratio and HDL-C /LDL-C risk ratio) while it was negatively correlated with HDL-C. This could be explained by the findings of Yi *et al*. which revealed that the antiapoptotic, proangiogenic and cardioprotective actions of CTRP-3 is significantly inhibited in the presence of CAD [21]. Moreover, this explains our results, the decreased levels of CTRP3 in female patients suffering from CAD secondary to long standing T2D when comparing to T2D alone. This novel finding highlights the possible use of CTRP3 as a diagnostic and prognostic marker in the development of CAD in female patients with long standing T2D.

In this study CTRP9 showed decreased serum levels in females suffering from CAD, T2D and CAD secondary to T2D. Our results indicated that CTRP9 exhibited a negative association with BMI, FBG, HbA1c %, insulin, HOMA-IR, TC, TG, LDL-C, TC/HDL-C and LDL-C /HDL-C, while it was positively associated with HDL-C, QUICKI, CTRP3 and MCP-1/CCL2. In addition, when using the multiple linear regression CTRP9 remained significantly correlated with LDL-C, FBG, BMI, T2D duration. To the best of our knowledge, this is the first study to correlate CTRP9 with T2D duration, QUICKI, CTRP3 and MCP-1/CCL2, there are no available data to concur or contradict our results. Surprisingly, CTRP9 was significantly negatively associated with CTRP3. These findings could be explained as the decreased CTRP9 levels in females suffering from T2D with or without CAD suggested that like APN in the absence of insulin, CTRP9 had little effect in suppressing gluconeogenesis [22], in contrast to CTRP3, which acted independently off insulin levels.[19] Moreover, the state of chronic low grade inflammation associated with increased dyslipidemia resulted in decreased levels of APN [23], consequently reducing the secretion of CTRP9 as it requires APN for its production [24].

As for the sera level of the proinflammatory chemokine MCP-1, it was found to be highest in female patients with CAD secondary to T2D, followed by patients with CAD, then T2D only, on comparison with the control group. Our results showed that MCP-1 was positively associated with BMI, FBG, HbA1c %, insulin, TC, TG, LDL-C, TC/HDL-C, LDL-C /HDL-C and HOMA-IR, while negatively associated with age, T2D duration, HDL-C, QUICKI and CTRP9. Another novel finding in this study, was the negative correlation between MCP-1 and CTRP9, which proposes that the anti-inflammatory action of CTRP9 results from reducing MCP-1 production. This could be attributed to the close similarity between CTRP3 and CTRP9, as both markers are the closest members to APN within the CTRP superfamily [9,11] and could be explained by Juskewitch *et al*. that MCP-1 was the only predictive cytokine of early systemic inflammation which resulted from inflammatory pathways such Lipopolysaccharide(LPS) induced inflammation [25], on which CTRP3, and as we proposed CTRP9, function as novel and endogenous antagonists in adipose tissue, thus blocking LPS induced inflammation and production of MCP-1[26]. These findings indicate a potential role for MCP-1 in combination with CTRP3 and CTRP9 as a diagnostic and prognostic tools in T2D and CAD as a complication of T2D.

## Conclusion

In conclusion, this study investigated the association of CTRP3, CTRP9 and MCP-1/CCL2 with hyperglycemia and the risk of development of CAD. This study also proposed a mechanism explaining the conflicting results of CTRP3 in T2D using the duration of T2D and a possible association between MCP-1/CCL2 and CTRP9. Accordingly, CTRP3 and CTRP9 could be potential markers recommended for the clinical use in the diagnosis, prognosis and follow up of patients with T2D at risk of developing CAD. Extension to this current study using a larger sample size, comparing newly diagnosed T2D patients with long standing T2D patients, should be done to support the previous findings.
